# Deep learning based CT images automatic analysis model for active/non-active pulmonary tuberculosis differential diagnosis

**DOI:** 10.3389/fmolb.2022.1086047

**Published:** 2022-12-05

**Authors:** Mayidili Nijiati, Renbing Zhou, Miriguli Damaola, Chuling Hu, Li Li, Baoxin Qian, Abudukeyoumujiang Abulizi, Aihemaitijiang Kaisaier, Chao Cai, Hongjun Li, Xiaoguang Zou

**Affiliations:** ^1^ Department of Radiology, The First People’s Hospital of Kashi Prefecture, Kashi, China; ^2^ Department of Colorectal Surgery, The Sixth Affiliated Hospital, Sun Yat-sen University, Guangzhou, China; ^3^ Department of Radiology, Beijing Youan Hospital, Capital Medical University, Beijing, China; ^4^ Huiying Medical Technology, Beijing, China; ^5^ Clinical Medical Research Center, The First People’s Hospital of Kashi Prefecture, Kashi, China

**Keywords:** active pulmonary tuberculosis, computer tomography image, machine learning, artificial intelligence, computer-aided diagnosis

## Abstract

Active pulmonary tuberculosis (ATB), which is more infectious and has a higher mortality rate compared with non-active pulmonary tuberculosis (non-ATB), needs to be diagnosed accurately and timely to prevent the tuberculosis from spreading and causing deaths. However, traditional differential diagnosis methods of active pulmonary tuberculosis involve bacteriological testing, sputum culturing and radiological images reading, which is time consuming and labour intensive. Therefore, an artificial intelligence model for ATB differential diagnosis would offer great assistance in clinical practice. In this study, computer tomography (CT) scans images and corresponding clinical information of 1160 ATB patients and 1131 patients with non-ATB were collected and divided into training, validation, and testing sets. A 3-dimension (3D) Nested UNet model was utilized to delineate lung field regions in the CT images, and three different pre-trained deep learning models including 3D VGG-16, 3D EfficientNet and 3D ResNet-50 were used for classification and differential diagnosis task. We also collected an external testing set with 100 ATB cases and 100 Non-ATB cases for further validation of the model. In the internal and external testing set, the 3D ResNet-50 model outperformed other models, reaching an AUC of 0.961 and 0.946, respectively. The 3D ResNet-50 model reached even higher levels of diagnostic accuracy than experienced radiologists, while the CT images reading and diagnosing speed was 10 times faster than human experts. The model was also capable of visualizing clinician interpretable lung lesion regions important for differential diagnosis, making it a powerful tool assisting ATB diagnosis. In conclusion, we developed an auxiliary tool to differentiate active and non-active pulmonary tuberculosis, which would have broad prospects in the bedside.

## 1 Introduction

Pulmonary tuberculosis (TB) is one of the top ten causes of death throughout the world and remains a major public health issue (Force et al., 2016; Ankrah et al., 2018). In 2020, 9.87 million cases of TB were recorded globally, with an incidence rate of 127 per 100,000. Among the 30 countries with a high TB burden, China ranks the second in the estimated pulmonary TB incidence after India (2.59 million) (Harding, 2020). Tuberculosis is caused by *Mycobacterium tuberculosis*, and most patients with *Mycobacterium tuberculosis* infection are asymptomatic, which is known as latent tuberculosis infection. Without timely and proper treatment, 10% of patients with latent infection will progress to active pulmonary tuberculosis (ATB), whose mortality rate is up to 50% at present (Klann et al., 2019). According to statistics, there are about 20 million existing active TB cases worldwide, and about 8–10 million new cases of TB are diagnosed each year (Zhu et al., 2020). Tuberculosis occurs in every corner around the globe, while Asia is the place with the highest incidence of tuberculosis, accounting for 60% of the global incidence (Ankrah et al., 2018; Collaborators, 2018). In clinical practice, there are still many challenges in the accurate detection and diagnosis of ATB. In different parts of China, for example, underdiagnoses and underreporting of TB is still prevalent in large general hospitals of eastern cities with better health care services, and the scarcity of medical resources in the rural areas of central and western regions leads to even lower TB detection rates and more serious underdiagnoses (Martini et al., 2020).

The gold standard for TB diagnosis is the TB smear and culture, but it takes a long time to finish a single test of *Mycobacterium tuberculosis* culture. To make it worse, the proportion of the bacillus-negative cases is as high as 30%–40%, implying that half of the patients with active TB are unable to achieve a definitive diagnosis of TB by means of *Mycobacterium tuberculosis* culture (Martini et al., 2020). In addition, molecular biology diagnostic techniques such as GeneXpert and TrueNAT have improved the speed and accuracy of diagnosing ATB to a large extent, but these tests increase the cost for diagnosis (Suen et al., 2015; Walzl et al., 2018). Therefore, cheaper and faster radiological imaging diagnostic methods are of great importance for the diagnosis of tuberculosis, such as chest radiography and computer tomography (CT). Despite of the fact that chest radiographs can be used to screen for TB, they are less sensitive and accurate than CT scans in terms of TB diagnosis, since CT scans are much more sensitive in the detection and characterisation of focal microscopic lesions in the lung, diffuse lesions in the lung parenchyma and mediastinal lymph node enlargement. However, the efficiency and accuracy of diagnosis based on CT scans are heavily dependent on the experience of the physician (Yang et al., 2019; Wang et al., 2021). The lack of physicians specialized in radiology makes it difficult to ensure the overall efficiency and accuracy of ATB diagnosis by CT images in clinical practice in many places. In addition, there are many different features and characteristics in the CT scan images of TB patients, which have proved to be either coexisting or interchangeable, making it more difficult to distinguish ATB. These obstacles pose a significant challenge to the physicians in terms of CT images reading, making the diagnosis of ATB a difficult and time-consuming task.

Recently, the application of radiomics (Lambin et al., 2012) and deep learning in TB diagnosis has shown good results, especially in utilizing the deep learning convolutional neural networks (CNNs). Li et al. used a 3D CNN model to distinguish patients with active TB from healthy individuals with an accuracy of 89.2% (Li et al., 2021). Wang et al. developed a 3D ResNet model for identifying non-tuberculous mycobacterial lung disease and *Mycobacterium tuberculosis*-like lung disease with an area under the receiver operating characteristic curve (AUC) value of 0.86 (Wang et al., 2021). Hwang et al. developed a deep learning automatic detection algorithm using chest radiographs, reaching a sensitivity of 94.3% and a specificity of 91% (Hwang et al., 2019). Khan et al. trained a CNN to calculate the probability of active and cured TB utilizing chest radiographs, and the AUC value of the model was higher than that of a pulmonologist (Khan et al., 2020). Yang et al. constructed a deep network model combining UNet and fast RCNN, which was able to detect and identify TB lesions and achieved significant performance (Yang et al., 2019). In short, deep learning techniques have been successfully applied to the analysis of the medical images, so in this context, automatic differential diagnosis of active and inactive TB disease based on deep learning will hold considerable promise.

In this study, we made use of 3D ResNet-50, a type of 3D CNN model, to distinguish active pulmonary tuberculosis from non-active pulmonary tuberculosis based on CT scans images. To the best of our knowledge, this was the first attempt utilizing 3D ResNet-50 for active pulmonary tuberculosis diagnosis. The automatic diagnosis model also provided class activation maps (CAMs) on the CT scan images to visualize the most indicative areas for diagnosis, allowing further interpretation into the artificial intelligence (AI) diagnosis models and physician-AI collaboration in the bedside. With a clinician-machine competition experiment, the 3D ResNet-50 ATB diagnosis algorithm was shown to have both human expert level diagnostic capabilities and fast diagnosis speed.

## 2 Materials and methods

### 2.1 Study setting and population

This study provided a retrospective analysis of pulmonary infection cases between January 2018 and December 2020 at the First People’s Hospital of Kashgar Region. The study was approved by the institutional review board of the participating hospitals, waiving the requirement of informed consent for each patient. Tuberculosis cases were classified as active TB cases or non-active TB cases according to the diagnostic criteria documented in the People’s Republic of China health industry standard “WS196-2017 Classification of Tuberculosis” (Liu and Zhou, 2018). Diagnostic criteria for active TB cases were as follows: 1) pathogenetically positive for *Mycobacterium tuberculosis*; 2) clinical signs and symptoms associated with TB, including cough, sputum, fever, night sweats, chest pain, and wasting; 3) imaging manifested by single or multiple manifestations such as solid lesions, nodules, caseous cavities, hairy glass density shadow, and tree-bud sign. Diagnostic criteria for non-active TB cases were as follows: 1) pathogenetically negative for *Mycobacterium tuberculosis*; 2) imaging manifestations include calcified lesions (single or multiple), cord-like lesions (clear margins), sclerotic lesions, clean cavities, pleural thickening, adhesions or calcifications, and other single or multiple manifestations. Patients with complete and clear chest CT scans images were enrolled in this study, while others with 1) incomplete clinical records, 2) concurrent conditions that severely affect the findings of chest CT (e.g., lung cancer, pacemaker or defibrillator placement, history of cardiac or pulmonary surgery) or 3) extensive pleural effusion were excluded.

**FIGURE 1:**
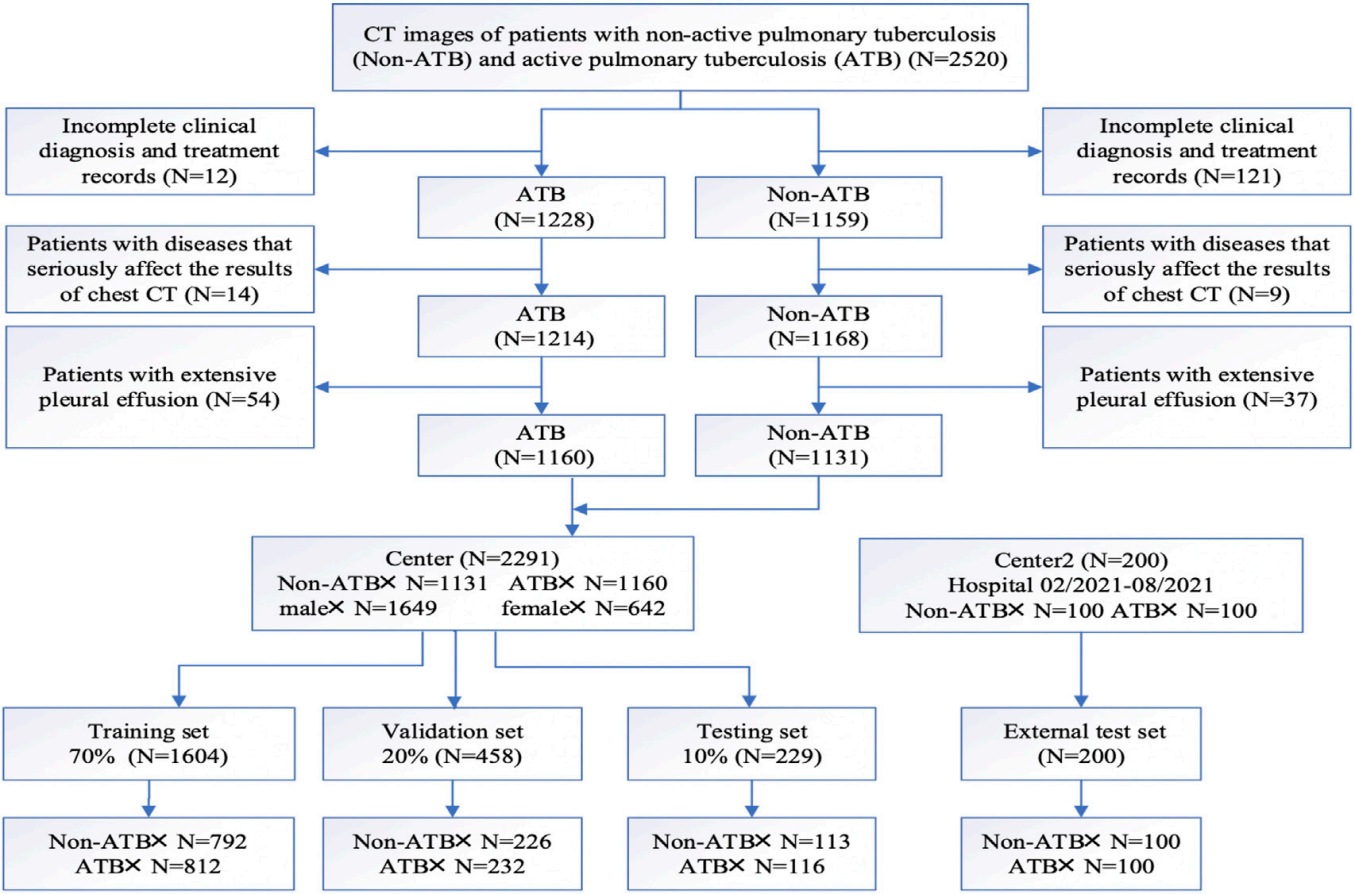
Patient inclusion and exclusion diagram.

According to the above criteria, a complete flow chart of the data collection was shown in Figure 1. A total of 2,291 patients were enrolled in this study, including 1,160 patients in the active pulmonary TB group (age: 54.62 ± 17.21 years; 280 female and 880 male) and 1,131 patients in the non-active pulmonary TB group (age: 45.12 ± 18.52 years; 362 female and 769 male). The patients’ demographic characteristics were listed in Table 1, including age, sex and existing TB related symptoms (chest pain, cough, sputum, fever, chest tightness, haemoptysis, wheezing, dyspnoea, fatigue, night sweats, and weight loss). CT images and clinical information were collected within 1 month before collecting samples for pathogenic microbiological examinations. Patients and their corresponding CT images and clinical data were randomly grouped at a ratio of 7:2:1, serving as training, validation and testing sets. Following the same criteria, we retrospectively collected 100 ATB patients and 100 non-ATB patients admitted to the Shache County Hospital of Kashgar Prefecture (Kashgar, China) between February and August in 2021 as an external testing set.

**TABLE 1:** Baseline characteristics of the 2291 patients.

Clinical features	Total (N = 2291)	Non-active pulmonary tuberculosis (N = 1131)	Active pulmonary tuberculosis (N = 1160)	*p* Value
Age	49.92 ± 17.41	45.12 ± 18.52	54.62 ± 17.21	<0.001
Sex				
Female	642 (28.02%)	362 (15.80%)	280 (12.22%)	0.079
Male	1649 (71.98%)	769 (33.57%)	880 (38.41%)	
Hemoptysis	274 (11.96%)	135 (5.89%)	139 (6.07%)	0.773
Cough	1901 (82.98%)	870 (37.97%)	1031 (45.00%)	0.023
Expectoration	1557 (67.96%)	757 (33.04%)	800 (34.92%)	0.702
Fever	1008 (44.00%)	441 (19.25%)	567 (24.75%)	0.098
Wheezing	274 (11.96%)	169 (7.38%)	105 (4.58%)	0.081
Chest pain	18 (0.79%)	13 (0.57%)	5 (0.22%)	0.01
Chest tightness	206 (8.99%)	113 (4.93%)	93 (4.06%)	0.627
Breathing difficulty	45 (1.96%)	11 (0.48%)	34 (1.48%)	0.474
Fatigue	458 (19.99%)	339 (14.80%)	119 (5.19%)	0.132
Night sweats	229 (10.00%)	101 (4.41%)	128 (5.59%)	0.613
Weight loss	457 (19.95%)	248 (10.82%)	209 (9.12%)	0.51

### 2.2 Computer tomography scan images collection

**FIGURE 2:**
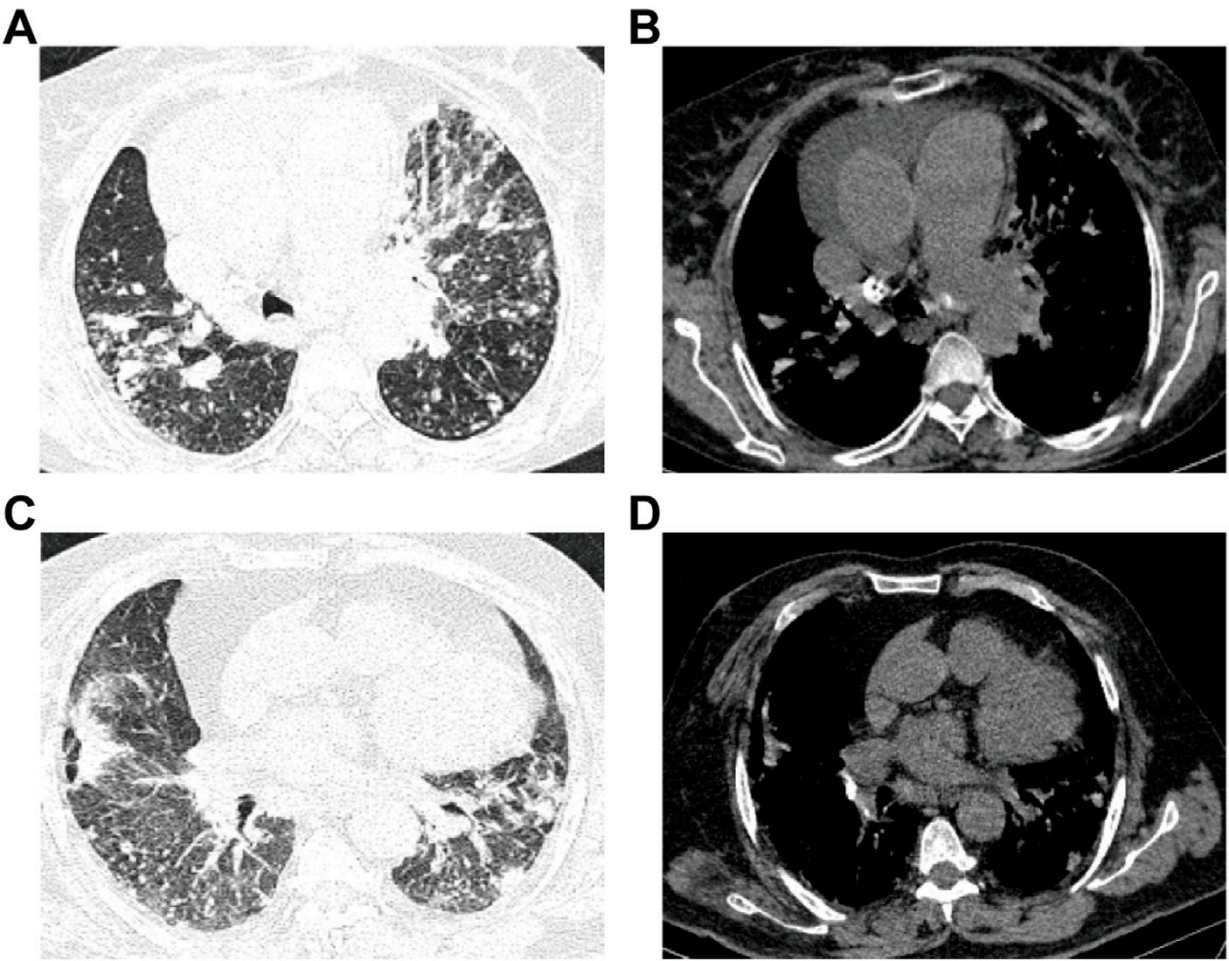
Images for ATB and non-ATB. **(A–B)** Patient with active pulmonary TB: male, 60-years-old, chest pain and dyspnea for 3 days, multiple central lobular nodules in the right lower lobe, left upper lobe and left lower lobe, and with cords and surrounding patchy dense shadowing. **(C–D)** Patient with non-active pulmonary TB: female, 50-years-old who experienced cough and sputum for 6 days, with multiple patchy shadows observed in the middle lobe of the right lung and the lower lobes of both lungs, adjacent to the pulmonary nodules and surrounded by tree buds.

CT scans were completed by using a Siemens CT scanner with a conventional layer thickness of 10 mm, a layer spacing of 10 mm, a thin layer thickness of 2–3 mm, a layer spacing of 2–3 mm, a table feed speed of 5 mm/s, a voltage of 120 kV, and a current of 180–240 mA. For the procedure, the patient was placed in the supine position with the arms raised, and the entire lung was scanned with a layer thickness and layer spacing of 5 mm each, followed by post-reconstruction with a layer thickness of 0.625 mm. The images were checked through both the lung window and the mediastinal window to ensure the quality of the images (Figure 2).

### 2.3 Development and comparison of the pulmonary tuberculosis differential diagnose models

**FIGURE 3:**
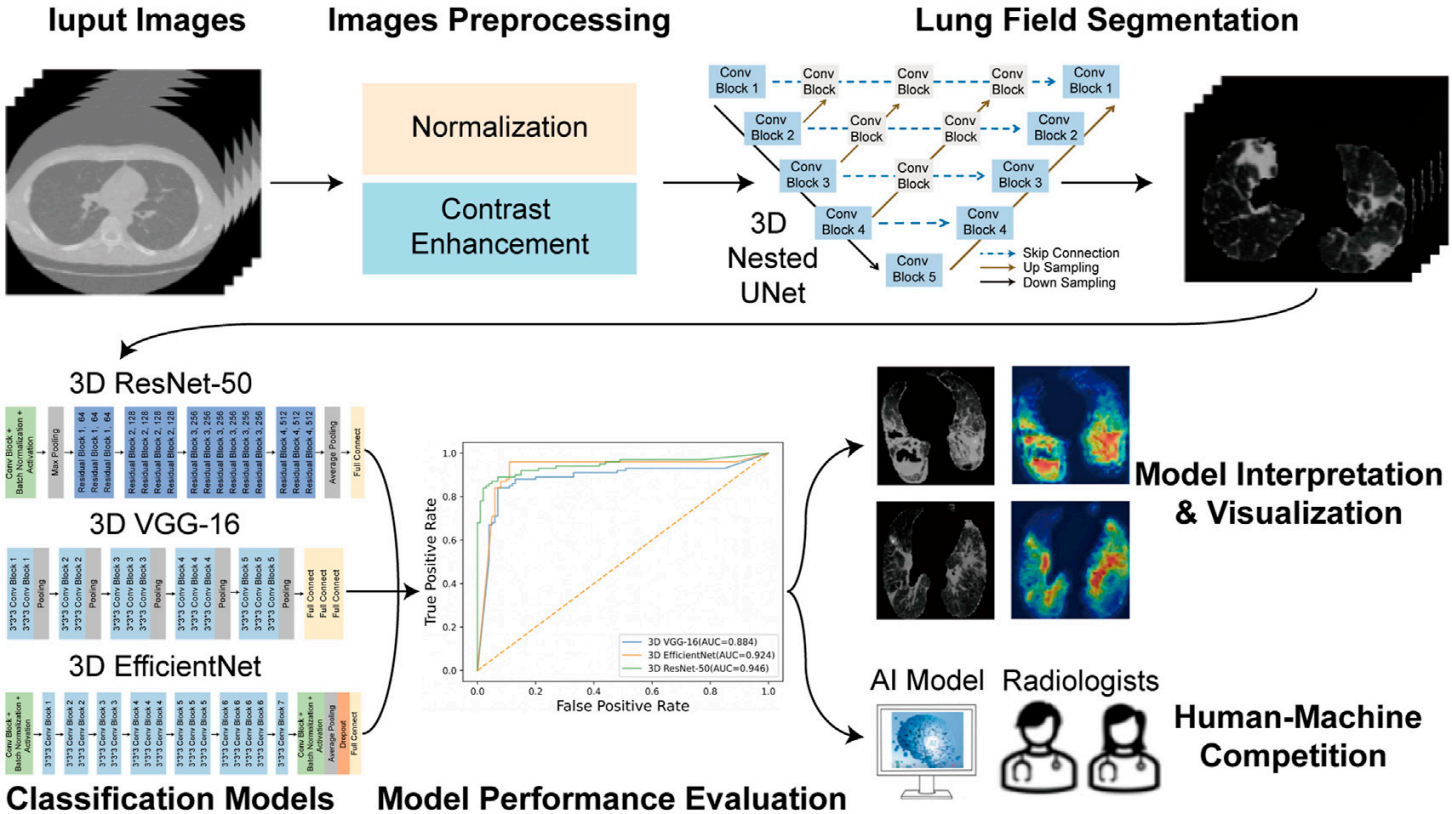
Overall flowchart of the study.

**FIGURE 4:**
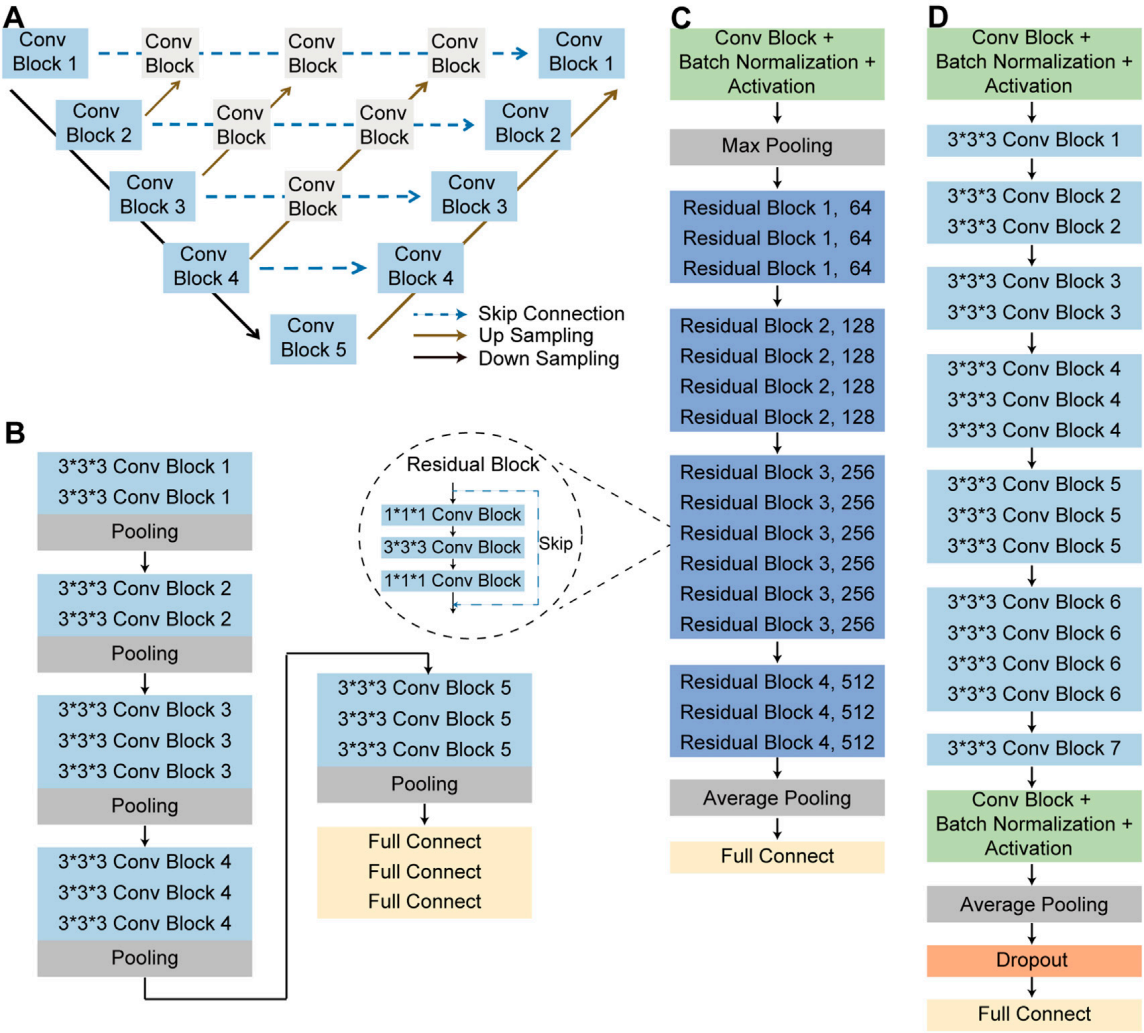
The structure of neural networks. **(A)** Nested UNet network structure. **(B)** 3D VGG-16 network structure. **(C)** 3D ResNet-50 network structure. **(D)** 3D EfficientNet network structure.

The overall flowchart of this study was shown in Figure 3. The whole process was divided into two parts. In the beginning of the first part, image preprocessing, such as normalization and contrast enhancement (Supplementary File S1), was finished. Then, in order to generate masks of the lung so as to eliminate the unrelated regions before the training of the deep learning model, we used 3D Nested UNet (Zhou et al., 2018) to extract of the lung field. To verify the effectiveness of the pretrained lung field segmentation model, we invited two radiologists with more than 5 years working experience to conduct a double-blind review of the segmentation results and to modify them to obtain the final segmentation results. The network structure was shown in Figure 4A and the details of 3D Nested UNet model saw Supplementary File S2.

To complete the classification task and achieve a superior classification performance, the second part used three different pre-trained deep learning models, including 3D ResNet-50 (Korolev et al., 2017), 3D VGG-16 (Apostolopoulos and Mpesiana, 2020) and 3D EfficientNet (Tan and Le, 2019). These AI models were widely used in the field of target classification, often as part of the classical neural network of the computer vision task backbone. The 3D VGG-16, 3D ResNet-50, and 3D EfficientNet were three dimensions modified version of the traditional two dimensions-VGG-16 network, two dimensions-residual Net network and two dimensions-EfficientNet network. 3D VGG-16, as the name implies, was consisted of 16 layers, including 13 convolutional layers and 3 fully connected (FC) layers, whose network structures was shown in Figure 4B. Using skip connections and residual blocks structure, 3D ResNet-50 model could improve learning efficiency and alleviate the problem of vanishing gradient caused by deep neural network. The network structure of 3D ResNet-50 model was shown in Figure 4C and the corresponding details of the model was described in Supplementary File S3. As for the third model, 3D EfficientNet network aimed at improving diagnosis performance by changing and balancing the depth of the feature extraction layers and the width of the deep learning model, and its network structures was shown in Figure 4D. These trained models utilized the same training, validation, testing and external testing sets. Training parameters were described in Supplementary File S4. After model training, we compared the performance of these three models in the training, validation, testing and external testing sets. The accuracy (ACC), the recall (also called the true positive rate or sensitivity), F1 score and the AUC value were calculated and used as evaluation indexes for performance comparison among different models.

### 2.4 Model interpretation and visualization

To visualize the inference process of the deep learning model, class activation maps (CAMs) were utilized to display the suspicious lung regions recognized by the deep learning model. The multi-image 3D CT scans were fed into the trained model, and then features from the final convolutional layer were extracted to generate a weighted activation map for each image *via* the 3D gradient-weighted class activation map (Grad-CAM) technique (Selvaraju et al., 2017) (Details in Supplementary File S5). CAMs enabled the predicted class scores to be visualized on any given image, highlighting the most discriminative and important regions for differential diagnosis with red and yellow masks to improve the interpretability of the model.

### 2.5 Human-machine diagnosis ability competition

At last, using the external test data, we supplemented a human-machine competition experiment to evaluate the total diagnostic time and accuracy between two radiologists and the best-performing AI model. We enlisted two radiologists with 5 and 10 years of professional experience who were not involved in annotating either the training or validation set. The CT scans reading rules were as follows: each radiologist interpreted the CT signs and calculated the proportion of patients with certain CT signs in their respective groups. During the study, each radiologist gave his own independent diagnosis for each case included in the external testing set.

### 2.6 Statistical analysis

SPSS version 22.0 (SPSS Inc) was used to analyse the differences between the clinical features of the active pulmonary TB group and the non-active pulmonary TB group. Continuous variables were expressed as the mean ± standard deviation, and a two-sided Student’s t test was used to identify significant differences between the variables in the different groups. Discrete variables were expressed as counts (percentages), and Pearson’s chi-square test was used for these variables. The comparison of predictive performances between multiple models was performed by the Delong test, and *p* values were computed using a one-tailed z-test. McNemar test was used to compare the differences of the sensitivity and specificity in ATB diagnosis between the AI model and radiologists. *p* values <0.05 were considered significant.

## 3 Results

### 3.1 Clinical characteristics of the patients

A total of 2,291 patients from The First People’s Hospital of Kashi were enrolled in this study, including 1160 patients with active pulmonary TB and 1,131 patients with non-active pulmonary TB. The patients’ demographic characteristics were listed in Table 1, active pulmonary TB patients were older than non-active pulmonary TB patients (*p* < 0.001). Patients with active pulmonary TB had a higher incidence of cough (*p* = 0.023), while patients with non-active pulmonary TB had a higher incidence of chest pain (*p* = 0.010). Patients and their corresponding CT images and clinical data were randomly grouped at a ratio of 7:2:1, serving as training set, validation set and testing set. In terms of the external testing cohort, 100 ATB patients and 100 non-ATB patients were collected in the Shache County Hospital of Kashgar Prefecture.

### 3.2 Deep learning models performance evaluation

**FIGURE 5:**
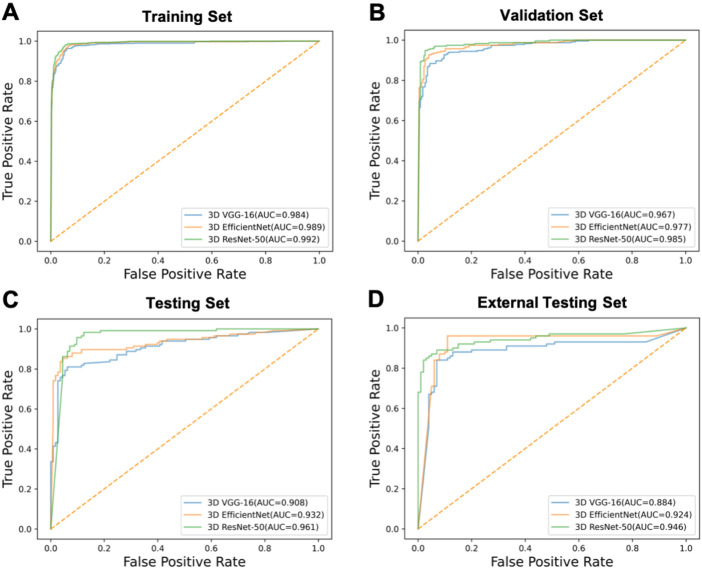
The ROC curves of the models. ROC curves of 3D VGG-16, 3D EfficientNet and 3D ResNet-50 models on the **(A)** training, **(B)** validation, **(C)** testing and **(D)** external testing sets.

We compared the performance of 3D ResNet-50 with other deep learning models including 3D VGG-16 and 3D EfficientNet architectures on the training, validation, testing and external testing sets. The results were presented in Table 2; Table 3. The results unveiled that the classification performance for 3D ResNet-50 was the highest in the training set, validation set and testing set. 3D ResNet-50 model achieved an AUC of 0.961 and an accuracy of 0.971 in the testing set, outperforming the other two models (3D ResNet-50 AUC vs. 3D VGG-16 AUC: 0.961 vs. 0.908, Delong test *p* = 0.028; 3D ResNet-50 AUC vs. 3D EfficientNet AUC: 0.961 vs. 0.932, *p* = 0.017). 3D ResNet-50 also had superior diagnosis capability over the 3D VGG-16 model and 3D EfficientNet model in the external validation set (3D ResNet-50 AUC vs. 3D VGG-16 AUC: 0.946 vs. 0.884, Delong test *p* = 0.012; 3D ResNet-50 AUC vs. 3D EfficientNet AUC: 0.946 vs. 0.924, *p* = 0.032). The ROC curves of the three models on the training, validation, testing and external testing sets were depicted in Figure 5, suggesting that 3D ResNet-50 is the model with best performance. These results showed that an appropriate increase of the depth of the neuron network according to the specific task could improve the classification performance of the deep learning model and avoid the excessive expansion of the complexity of the model, which might result in diminishing the capabilities of the model in identifying active pulmonary tuberculosis cases.

**TABLE 2:** Performance of 3 different deep learning models on the training, validation, testing and external testing sets.

	Training set			Validation set			Testing set			External testing set		
	VGG-16	EfficientNet	ResNet-50	VGG-16	EfficientNet	ResNet-50	VGG-16	EfficientNet	ResNet-50	VGG-16	EfficientNet	ResNet-50
AUC (95%CI)	0.984	0.989	0.992	0.967	0.977	0.985	0.908	0.932	0.961	0.884	0.924	0.946
	(0.979–0.990)	(0.984–0.993)	(0.988–0.995)	(0.952–0.981)	(0.965–0.989)	(0.975–0.995)	(0.869–0.948)	(0.897–0.967)	(0.935–0.987)	(0.830–0.938)	(0.880–0.969)	(0.911–0.981)
ACC	0.987	0.992	0.996	0.961	0.978	0.989	0.912	0.944	0.971	0.89	0.905	0.91
Recall	0.944	0.967	0.977	0.922	0.943	0.955	0.898	0.91	0.935	0.88	0.88	0.89
FI-score	0.938	0.958	0.962	0.914	0.931	0.944	0.886	0.903	0.924	0.889	0.904	0.908

**TABLE 3:** Comparison of 3 different deep learning models on the training, validation, testing and external testing sets.

Models for comparison	P_Trainning Set_	P_Validation Set_	P_Testing Set_	P_External Testing Set_
3D VGG-16 vs. 3D EfficientNet	0.012	0.034	0.046	0.016
3D EfficientNet vs. 3D ResNet-50	0.021	0.045	0.017	0.032
3D VGG-16 vs. 3D ResNet-50	<0.001	0.013	0.028	0.012

### 3.3 Model visualization

**FIGURE 6:**
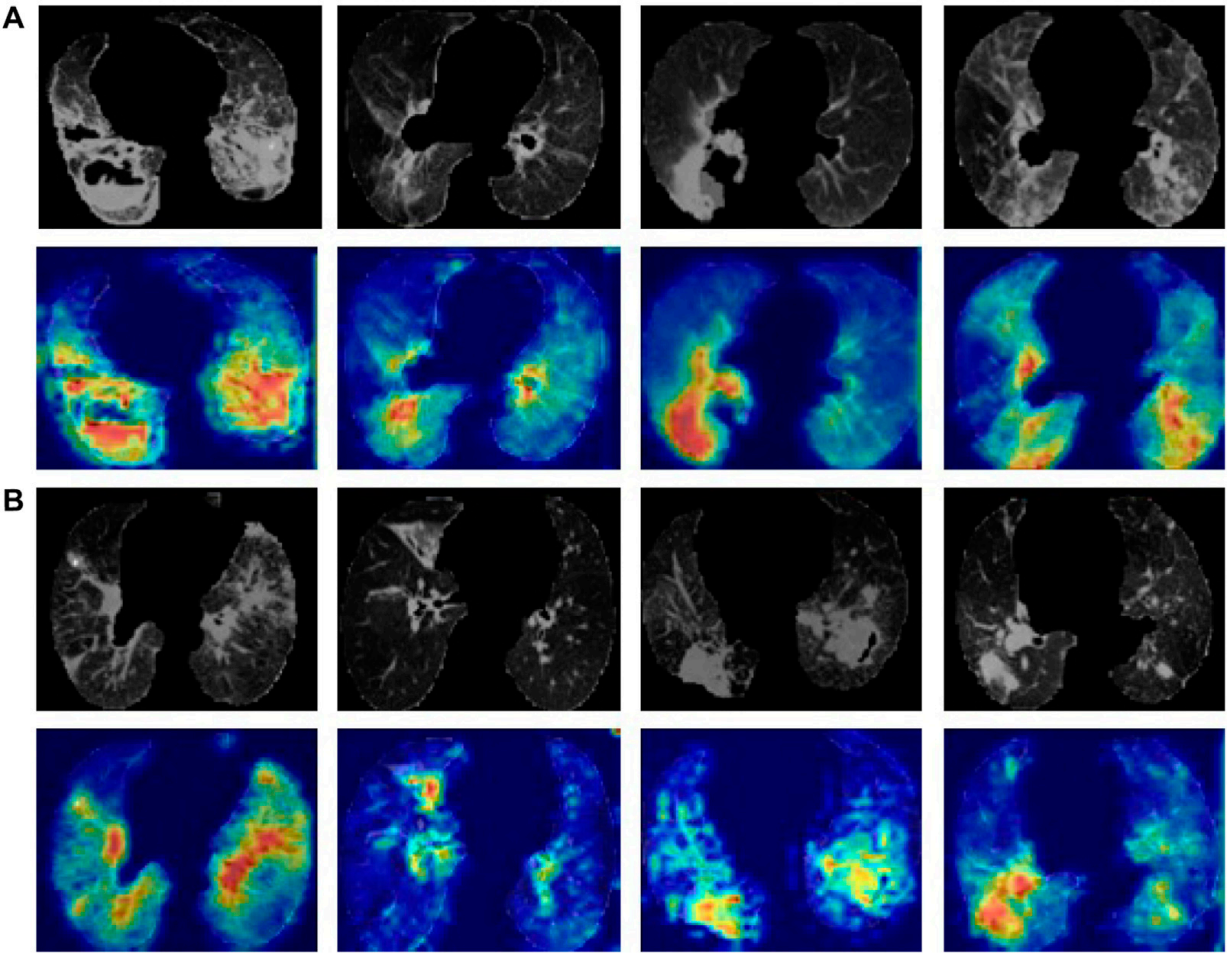
Lung CT images and the corresponding CAMs. **(A)** ATB CT images of the lung field and the corresponding CAMs. **(B)** Non-ATB CT images of the lung field and the corresponding CAMs.

To visualize the inference process of a deep learning model, we selected the model with the best performance for feature visualization using CAMs, masking the most representative regions of the predicted disease on the CT scan images with red colour. The Grad-CAM tool was applied to identify and visualize the regions that the 3D ResNet-50 model considered important for differential diagnosis. Figure 6A showed the lung field region CT images and the corresponding CAMs of four active pulmonary TB patients, and Figure 6B showed the lung field region CT images and the corresponding CAMs of four non-active pulmonary TB patients. The high attention and high activation areas shown by the CAMs are tuberculosis affected regions with vital information for the ATB/non-ATB differential diagnosis, indicating that the AI model did not only provide us with accurate diagnosis but also focused on the important lung lesion regions when it was reading the CT images, imitating the behaviour of radiology experts. Obviously, it would greatly boost the clinicians’ confidence in the AI diagnosis, which might become a powerful tool in the bedside.

### 3.4 The comparison between AI model and radiologist in active pulmonary tuberculosis diagnosis

In the external testing set, the total diagnostic time and accuracy between two radiologists and the best-performing AI model (3D ResNet-50) were compared. The results are summarized in Table 4. Classification confusion matrices reported the number of true-positive, false-positive, true-negative and false-negative results of the AI model and radiologists. The 3D ResNet-50 model achieved an ACC of 0.910 in distinguishing active pulmonary TB from non-active pulmonary TB, reaching higher levels of sensitivity and sensitivity attained by both human experts, including radiologist with 10 years of experience (3D ResNet-50 vs. radiologist sensitivity: 0.894 vs. 0.876, McNemar test *p* < 0.05; 3D ResNet-50 vs. radiologist specificity: 0.927 vs. 0.915, McNemar test *p* < 0.05) and radiologist with 5 years of experience (3D ResNet-50 vs. radiologist sensitivity: 0.894 vs. 0.863, McNemar test *p* < 0.05; 3D ResNet-50 vs. radiologist specificity: 0.927 vs. 0.888, McNemar test *p* < 0.05). Additionally, the speed of diagnosis was 10 times faster than that of the radiologists. In other words, the AI model was capable of producing experienced radiologist-level diagnosis rapidly and automatically, which would improve the working efficiency of the physicians and address the problem of the lack of experienced radiologists.

**TABLE 4:** Experienced radiologist-machine diagnosis ability competition in the external testing set.

	3D ResNet-50		Radiologists-10years		Radiologists-5years	
	ATB	Non-ATB	ATB	Non-ATB	ATB	Non-ATB
Predicted ATB	93	7	92	8	88	11
Predicted Non-ATB	11	89	13	87	14	87
Total time	5min		48min		52min	
Accuracy (ACC)	0.91		0.895		0.875	

## 4 Discussion

In this study, we established CT scans-based AI models for the active tuberculosis diagnosis. Among the deep learning models, the 3D ResNet-50 model outperformed the 3D VGG-16 model and 3D EfficientNet model, reaching AUC values over 0.94 in multiple cohorts. It was worth mentioning that the diagnosis speed of the 3D ResNet-50 model was 10 times faster than that of the radiologists, while the accuracy was about the same as experienced radiologists. Clinically, distinguishing patients with non-active pulmonary TB from patients with active pulmonary TB based solely on CT images is challenging because CT images of both conditions show multiple nodules, funicular foci, patchy dense shadows, and buds. Here, the regions highlighted in the images by the CAMs could assist radiologists in reading CT scans, which would maximize the working efficiency of the doctors and shorten the time required for the differential diagnosis.

Basic clinical data and clinical manifestations of all the enrolled patients were collected for further investigation. Patients of active pulmonary tuberculosis were previously reported to be older (Perez-Guzman et al., 1999; Li et al., 2017) than those with non-active pulmonary tuberculosis and more likely to have symptoms such as cough (Alavi et al., 2014) and chest pain (Kwon et al., 2013; Lee et al., 2021), which was consistent with the findings of our study. However, there was no difference in gender between active and non-active pulmonary tuberculosis patients in this study, which agreed with the findings of Kim et al. (2014) and Wang et al. (2021). Cui et al. (2020) tried to use radiomics to distinguish pulmonary tuberculosis and lung cancer with CT images, but it took a long time to delineate the disease affected regions of interest in advance and the results were greatly affected by the manual delineation. In contrast, our approach used 3D Nested UNet to extract the lung field, which was an accurate and fully automatic segmentation algorithm, keeping the lung field visual information intact for deep learning. Ma et al. used manual labelling method as a step for the segmentation of TB affected regions (Ma et al., 2020), which was less robust and automatic than models fully depended on machine learning algorithms. In addition, the Ma et al. adopted a 2D classification network, but our classification network was based on 3D, which made full use of three-dimensional features of images. In a word, our deep learning model had significant advantages over the reported CT image-based TB diagnosis models, which would find wide clinical application as a reliable tool for active pulmonary tuberculosis diagnosis.

Our study also had certain limitations. First, our cohort did not include TB patients with human immunodeficiency virus and patients with other subtypes of TB, such as drug-resistant tuberculosis and reactivated tuberculosis. Therefore, the diagnostic performance of the model for these patients remained unknown. Second, the model might be less sensitive to small pulmonary TB lesions. Therefore, physicians still need to check the CT scans again before coming up with the final diagnosis.

In conclusion, this study demonstrated that 3D ResNet-50 deep learning model was capable of achieving a diagnosis accuracy comparable to experienced radiologists and could be used as a rapid auxiliary diagnostic tool to differentiate active pulmonary tuberculosis and non-active pulmonary tuberculosis with indicative areas of the predicted disease visualized in the CT images.

## Data Availability

The clinical data and CT images are not publicly available for patient privacy protection purposes. Any individual affiliated with an academic institution may request access to the original images and clinical data from the corresponding author (Xiaoguang Zou) for non-commercial, research purposes.

## Nomenclature

### Abbreviations

AI: artificial intelligence
ATB: active pulmonary tuberculosis
AUC: area under the receiver operating characteristic curve
CAMs: class activation maps
CNN: convolutional neural networks
CT: computer tomography
non-ATB: non-active pulmonary tuberculosis
ROC: receiver operating characteristic curve
TB: tuberculosis
3D: three dimension
