# Supplementary material for: Deep learning based CT images automatic analysis model for active/non-active pulmonary tuberculosis differential diagnosis
Source: Front Mol Biosci. 2022 Dec 5;9:1086047. doi: 10.3389/fmolb.2022.1086047 (PMC9760807; doi:10.3389/fmolb.2022.1086047)
Supplement: Supplementary file 1 [file DataSheet1.docx]

**Supplementary file**

### **S1 Appendix. Image preprocessing**

The standard storage format of the raw CT image data involved the use of Digital Imaging and Communications in Medicine (DICOM). Before being input into the algorithm, the raw data were converted to a uniform format, and the dataset was cleaned to ensure that no erroneous data were included in the dataset, which would interfere with the experimental results. Additionally, to obtain a more robust model, we initially normalized the CT images by adjusting the window width and window position to [-1200, 600] and by normalizing the image intensity to [0,1]. The lung field segmentation grid was segmented using DICOM images of their original sizes. Due to differences in the patients’ body shapes, the volumes of lung fields segmented by different patients were quite different. When considering the computational constraints, all of the CT image data were normalized to 256×256×64 and sent to the classification network for training.

### **S2 Appendix. Lung field segmentation algorithm**

The network structure of 3D Nested UNet model consists of an encoder, decoder, dense skip path, and deep supervision. The encoder is used to extract the image features. This project uses a residual block structure to implement feature extraction. The use of this structure can optimally solve the problem of gradient attenuation with deepening of the network and can enable the deep network to be trained successfully. The decoder is used to fuse features and to restore the segmentation results. The dense connection is used to recover the features through skip connections to strengthen the learning of the features. Finally, the deep supervision structure can accelerate the convergence of the network. On this basis, to further improve the segmentation accuracy of the network, we improved the model from the perspective of the model itself by using weight attenuation for the convolutional layer, by adding a regularization term to the objective function, and by limiting the number of weight parameters to prevent overfitting. The dropout mechanism and batch normalization were used to render the data distribution more uniform and to suppress activation of all of the neurons in the convolutional layer, thus further improving the model fitting ability. We segmented lung fields in a 3D Nested UNet network based on the pretrained public dataset (Finding and Measuring Lungs in CT Data, https://www.kaggle.com/kmader/finding-lungs-in-ct-data).

### **S3 Appendix. Deep learning classification algorithm**

A deep residual network is designed to overcome the problems of low learning efficiency and ineffective improvement of accuracy caused by increasing network depth. The ResNet model is used to realize image classification, which has been widely used for its excellent module structure and high efficiency.ResNet is divided into five stages, where Stage 0 has a relatively simple structure and can be regarded as the preprocessing of input. The last four stages are composed of bottlenecks and have similar structures. Stage 1 contains three blocks, and the remaining three stages contain four, six, and three blocks. After five stages of processing, the classification results were obtained. The 3D Resnet-50 (50 represents 50 levels) network used in this paper is a typical residual network. Skip connections in the figure are residual connections, which can reinject earlier information into downstream data, alleviating the loss of information in upper-lower transmission to some extent, thus reducing the training difficulty of deep models. A model can map data records in a database to a given category, which can be applied to data prediction.

**S4 Appendix. Training parameters**

For model training, the Adam optimizer was used to train the network with a batch size of 2. The initial learning rate was set to 0.001 and every 100 epochs with a decay factor of 0.5. An early stopping criterion was used to terminate training due to the absence of further improvement in both loss and accuracy, and the model with the lowest validation loss was selected. During the training phase, the dropout strategy on the fully connected layers with a probability of 0.5 and the L2 regularization strategy on weight and bias were used to prevent the overfitting problem. All were run in Python version 3.6.8. We used the PyTorch framework to train the model on two NVIDIA RTX 1080 Ti graphics processing units for up to 4,500 iterations.

**S5 Appendix. Model visualization technology**

At present, deep neural networks have achieved remarkable performance in many visual tasks; however, deep neural network methods are different from other feature engineering methods. Specifically, convolutional neural networks are more similar to a black box. In this paper, we used a class activation map (CAM) to display the suspicious lung regions to demonstrate the inference process of a deep learning model in a visual manner.

The operation of CAM is defined by the following:

|  | $L_{Grad-CAM}^{c}=ReLU(\sum_{k} \alpha_{k}^{c}A^{k})$ | (1) |
| --- | --- | --- |

*A* represents the output of the final convolutional layer, *k* represents the *k^th^* channel in feature layer A, where $A^{k}$ represents the data of channel *k* in feature layer *A*, *c* is the number of categories, and $\alpha_{k}^{c}$ is the weight connecting.$A^{k}$. The heatmap $L_{Grad-CAM}^{c}$ is generated by a weighted combination of forward activation maps followed by a *ReLU*. Finally, by simply upsampling the class activation map to the size of the input image, we can indicate the discriminative image regions for a specific disease.The feature map required to generate the class mapping was extracted from the final convolutional after the images passed through the fully trained network. Heatmaps were produced using the packages Matplotlib and OpenCV.
